# Early Prevention of Atherosclerosis: Detection and Management of Hypercholesterolaemia in Children and Adolescents

**DOI:** 10.3390/life11040345

**Published:** 2021-04-14

**Authors:** Cristina Pederiva, Maria Elena Capra, Claudia Viggiano, Valentina Rovelli, Giuseppe Banderali, Giacomo Biasucci

**Affiliations:** 1 Clinical Service for Dyslipidaemias, Study and Prevention of Atherosclerosis in Childhood, Pediatrics Unit, ASST-Santi Paolo e Carlo, 20142 Milan, Italy; cristina.pederiva@asst-santipaolocarlo.it (C.P.); claudia.viaggiano@unimi.it (C.V.); valentina.rovelli@asst-santipaolocarlo.it (V.R.); giuseppe.banderali@asst-santipaolocarlo.it (G.B.); 2 Centre for Paediatric Dyslipidaemias, Paediatrics and Neonatology Unit, Guglielmo da Saliceto Hospital, 29121 Piacenza, Italy; G.Biasucci@ausl.pc.it

**Keywords:** hypercholesterolaemia, children, adolescents, early detection, CHD prevention

## Abstract

Coronary heart disease (CHD) is the main cause of death and morbidity in the world. There is a strong evidence that the atherosclerotic process begins in childhood and that hypercholesterolaemia is a CHD major risk factor. Hypercholesterolaemia is a modifiable CHD risk factor and there is a tracking of hypercholesterolaemia from birth to adulthood. Familial hypercholesterolaemia (FH) is the most common primitive cause of hypercholesterolaemia, affecting 1:200–250 individuals. Early detection and treatment of hypercholesterolaemia in childhood can literally “save decades of life”, as stated in the European Atherosclerosis Society Consensus. Multiple screening strategies have been proposed. In 2008, the American Academy of Pediatrics published the criteria for targeted screening, while some expert panels recommend universal screening particularly in the young, although cost effectiveness has not been fully analysed. Blood lipid profile evaluation [total cholesterol, Low-Density Lipoprotein Cholesterol (LDL-C), High-Density Lipoprotein Cholesterol (HDL-C) and triglycerides] is the first step. It has to be ideally performed between two and ten years of age. Hypercholesterolaemia has to be confirmed with a second sample and followed by the detection of family history for premature (before 55 years in men and 60 years in women) or subsequent cardio-vascular events and/or hypercholesterolaemia in 1st and 2nd degree relatives. The management of hypercholesterolaemia in childhood primarily involves healthy lifestyle and a prudent low-fat diet, emphasising the benefits of the Mediterranean diet. Statins are the cornerstone of the drug therapy approved in USA and in Europe for use in children. Ezetimibe or bile acid sequestrants may be required to attain LDL-C goal in some patients. Early identification of children with severe hypercholesterolaemia or with FH is important to prevent atherosclerosis at the earliest stage of development, when maximum benefit can still be obtained via lifestyle adaptations and therapy. The purpose of our review is to highlight the importance of prevention and treatment of hypercholesterolaemia starting from the earliest stages of life.

## 1. Introduction

**Figure 1:**
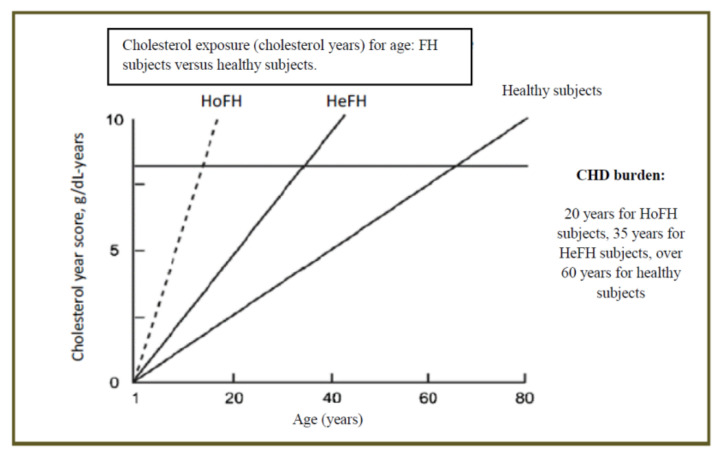
Cholesterol exposure per year and correlation to age onset of CHD, Modified from A. Wiegman [7].

Coronary Heart Disease (CHD) is the main cause of death and disability worldwide [1]. Preventive strategies in the adult population have led to a reduction in CHD mortality, even if the rate of mortality is still high. Numerous studies [2,3] have widely demonstrated that the atherosclerotic process dates back even to fetal age. Beginning in the fetus, the sustained exposure of the arterial wall to elevated Low-Density Lipoprotein cholesterol (LDL-C) accelerates cholesterol deposition and vascular inflammation, leading to arteries atherosclerosis and, eventually, to premature CHD [4]. Post-mortem studies [5] have demonstrated that fatty streaks are already present in childhood and that their extension is related to LDL-C levels. A strong relationship between non-HDL cholesterol levels and atherosclerotic lesions has been demonstrated both in pre-mortem (the Bogalusa heart Study) [5] and post-mortem (the Pathobiological Determinants of Atherosclerosis in Youth [PDAY] study) [6] studies [7]. A recent study has demonstrated that Carotidal Intima Media Thickness (cIMT) is higher in patients affected by familial hypercholesterolaemia (FH) compared to healthy subjects. The value of cIMT is directly related to LDL-C levels. In children with FH, cIMT is higher than those of non-affected siblings starting from eight years of age [8,9]. The exposure to LDL-C (expressed as increase in LDL-C grams per year) is directly linked to the so-called LDL burden in subjects with heterozygous FH (HeFH), in subjects with homozygous FH (HoFH) and in healthy subjects. Once the LDL burden has been reached, the occurrence of CHD is highly probable [7] (Figure 1). 

Early detection and treatment of children and adolescents with hypercholesterolaemia becomes therefore an issue of utmost importance for Pediatricians, because it enables to “save decades of life”, as stated by Wiegman in the European Atherosclerosis Society (EAS) guidelines [7]. An adequate, effective and early treatment of hypercholesterolaemia in terms of lifestyle modifications, acquisition of healthy eating habits and, if necessary, pharmacological treatment must begin in childhood. Starting such a treatment in childhood has the powerful effect of modifying atherosclerosis’ natural history, thus reducing CHD mortality and morbidity in adulthood [7,10]. The purpose of our review is to make paediatricians and physicians realise that hypercholesterolaemia is underdiagnosed and undertreated in childhood and adolescence; it must not be considered an issue involving only adult patients, but a complex process that starts even before birth. Therefore, the earlier we recognise and treat hypercholesterolaemia, the more “decades of life” we will be able to save. In this context, physicians dealing with children and adolescents are border troops: if they detect and treat atherosclerosis, many CHD battles will be spared in adulthood.

## 2. Dyslipidaemia in Childhood

In the “Effect of potentially modifiable risk factors associated with myocardial infarction in 52 countries Study”, known as INTERHEART study, 9 easily measured CHD risk factors have been identified: abnormal blood lipid levels, hypertension, diabetes, obesity, diet, physical activity, alcohol consumption, smoking and psycho-social factors [11]. These risk factors account for over 90% of the risk for acute myocardial infarction [11]. Dyslipidaemia can be defined as high levels of serum total cholesterol, elevated levels of LDL-C, elevated levels of triglycerides and/or low values of serum HDL-C [7,11]. Dyslipidaemia is an important CHD risk factor because it is detectable since the first stages of life and it is modifiable [7]. Moreover, there is a tracking of cholesterol levels from childhood to adult age. This means that a child with elevated blood lipid levels, if not detected and treated, will be an adult with elevated blood lipid values [7]. Dyslipidaemia in childhood can be primitive or secondary.

### 2.1. Primitive Forms of Dyslipidaemia

Primitive forms of dyslipidaemia are genetically determined and can be divided in monogenic and polygenic disorders [12]. Familial Hypercolesterolaemia (FH), with one rare recessive exception, is an autosomal dominant disorder [7]. FH results in reduced hepatic ability to clear LDL-C from the circulation, thus leading to increase of LDL-C in the blood vessels and LDL-C accumulation. HeFH is a common disorder, affecting approximately 1 out of 200–250 individuals in the general population, with a prevalence much higher than previously expected [7,13]. HoFH is a rare disease, with an estimated prevalence of 1 per 160,000–300,000 subjects [7]. FH can be caused by different gene mutations. The gene encoding the LDL receptor is the most frequently involved. Up to now, more than 1700 different mutations of the gene encoding LDL receptor have been detected. These mutations cause the absence of LDL receptors on the hepatocyte surface or the presence of a non-functioning LDL receptor, thus leading to an ineffective LDL clearance [14]. FH can be also due to a mutation in the Apolipoprotein B encoding gene (APOB gene). The Apolipoprotein B is a ligand for LDL receptor. This mutation occurs approximately in 5% of FH cases [14]. In 1% of cases, FH is caused by a mutation in Proprotein convertase subtilisin/kexin type 9 (PCSK9 gene), a protein that regulates the clearance of LDL receptors, modulating the number of LDL receptors present on the hepatocyte surface. Mutations of the Low Density Lipoprotein Receptor Adaptor Protein 1 (LDLRAP1 gene) are responsible for the recessive form of FH. In 5 to 30% of cases, FH can be caused by a mutation in unknown genes or may have a polygenic origin [14].

### 2.2. Secondary Forms of Dyslipidaemia

Secondary forms of dyslipidaemia can be caused by exogenous or endogenous factors [12]. Exogenous causes of dyslipidaemia include drug therapies with corticosteroids, isotretinoin, beta-blockers, some oral contraceptives, some chemotherapeutic agents and some antiretroviral agents. Alcohol abuse and excessive intake of saturated fatty acids are other exogenous causes of dyslipidaemia [12]. Dyslipidaemia can be also present as a consequence of a primitive endocrine, renal, infectious, inflammatory and cardiac disease. Endocrine diseases that can alter lipid profile include hypothyroidism, hypopituitarism, diabetes mellitus, polycystic ovary syndrome, lipodystrophy and acute intermittent porphyria. Renal diseases that can alter lipid profile include chronic renal disease, hemolytic uremic syndrome and nephrotic syndrome. Infectious diseases such as HIV and hepatitis, hepatic diseases such as obstructive liver disease, cholestasis conditions, biliary cirrhosis, Alagille syndrome and inflammatory disease such as systemic lupus erythematosus and juvenile rheumatoid arthritis can cause dyslipidaemia as well. Storage diseases like Glycogen-storage disease, Gaucher disease, Cystine-storage disease, Juvenile Tay-Sachs disease and Niemann-Pick disease and cardiac conditions like Kawasaki disease include in their clinical presentations an abnormal lipid profile [12].

## 3. Detection of Hypercholesterolaemia in Childhood

Early detection of children with severe hypercholesterolaemia is an issue of utmost importance in order to identify subjects at high cardiovascular risk [15]. It is increasingly recognised that childhood and early adolescence offer the most favorable timeframe for diagnosing FH as well as introducing and maintaining lifelong treatment and management strategies. To achieve such radical care from a young age, a shift in community and health professional perceptions of FH and its effects is required. Little attention has been given to data for FH screening in general practice and by paediatricians who can find most affected patients [16]. In countries with a history of dedicated screening programs, such as the Netherlands and Norway, the outcomes in terms of newly diagnosed FH index cases and cascade tested relatives are much higher than in countries lacking any formal screening program [13,17]. In Slovenia the use of universal screening for children aged above 5 years has been introduced to help the detection of FH, but feasibility and cost-effectiveness remain to be confirmed [18]. Selective screening of children with hypercholesterolaemia was first recommended in 1992, based on family history of premature CHD or hypercholesterolaemia [19]. Unfortunately, family history failed to identify the majority of children with FH [20,21] which led to the recommendation of a universal lipid screening program for children aged 9–11 years and young people aged 17–21 years [12]. In Canada, experts recommend a routine universal screening of plasma lipids at 11 years of age, or as early as 12 months in children with a first-degree family history of CHD or FH [22]. Wald et al. examined the efficacy and feasibility of child-parent screening for FH in primary care practices, at routine immunization attendances by children aged 1–2 years in the United Kingdom over a three-years period [23]. In a recent study we evaluated the knowledge of lipid and CHD issues in parents of newborns in two Italian hospitals: the percentage of adults who were unaware of their lipid profile, with a positive family history for CHD and/or lipid disorders was higher than expected and, as a result, even the number of undetected paediatric patients at high cardiovascular risk could be greater than expected [24]. Improving public awareness of the possibility of FH, especially in the community setting, needs to be addressed [25]. Many families may be aware of premature CHD death in their own households but the significance of these past events and the potential future risk for their own health is often not fully grasped. Young off-spring of affected patients are likely to feel entirely healthy and lacking in symptoms and see no reason to start life-long treatment for a condition they perceive as posing no immediate or potential threat. In this context, all screening strategies to identify children with FH and initiate early lipid management are characterised by low adherence by the medical community and limited compliance by parents and children [16]. In recent years, in many countries FH Registries have been instituted with the aim of generating large scale robust data on how FH is detected and managed. The potential of FH Registries and improved coding for FH needs to be linked to screening approaches, establishment and harmonization of the clinical diagnosis [25]. In Italy the LIPid transport disorders Italian GEnetic (LIPIGEN) Network connects Centres specialized in detection and treatment of subjects with primitive forms of dyslipidaemia, especially patients with FH, encouraging information sharing and creating consensus and recommendation papers so as to uniform clinical practice all over Italy [26,27,28]. In the European Atherosclerosis Society Consensus published in 2015 it is stated that screening for FH in children should be country specific, utilising all existing screening strategies, including opportunistic screening in the setting of a positive family history. Cascade screening based on genetic testing is advisable in settings available. Compared with universal screening, lipid screening in patients who have a known causative mutation, together with cascade testing of immediate family and relatives using DNA information, is very cost-effective, as 50% of them inherit the mutation [7]. Universal screening can be taken into account starting from 10 years of age in countries where this is feasible, especially where founder effects with markedly increased frequency of FH are prevalent, as in Quebec (Canada), South Africa and Lebanon [7]. Screening strategies for FH in children and adolescents are summarised in Table 1.

## 4. Diagnosis

FH may be suspected in children in at least three situations: a child from a family where HeFH has been identified or suspected (clinical/genetic criteria); a child from a family with a history of premature (before age 55 years in men and 60 years in women) CHD or a child from one or both parents displaying primary hypercholesterolaemia. This emphasises the importance of assessing the family history regarding cholesterol levels, CHD and confirmed or suspected conditions in all children [7]. The Dutch Lipid Clinic criteria and the Simon Broome criteria that have specific LDL cholesterol cut-offs for this age group, overall showed a poorer performance [29]. Any child with LDL-C ≥ 190 mg/dL on two consecutive occasions has a high probability of FH [7]. An LDL-C ≥ 160 mg/dL in a child with a family history of premature CHD in a close relative and/or baseline high cholesterol in one parent indicates a high probability of FH. If the parent has a genetic diagnosis of FH, an LDL-C ≥ 130 mg/dL in the child suggests FH [7]. Children above 5 years of age should be offered testing when a parent (or close relative) is identified with FH [7]. As first screening test, a non-fasting lipid profile is sufficient, but LDL-C levels should be measured at least twice over 3 months in a fasting state to confirm the diagnosis [30]; it is advisable to evaluate blood levels of total cholesterol, LDL-C, HDL-C and triglycerides. If non-HDL levels are higher than 145 mg/dL in a non-fasting lipid profile, a fasting lipid profile determination is advisable [12,30]. The optimal window for screening is between 2 and 10 years of age, in order to to minimise the effect of pubertal activation and diet. Below two years of age lipid values present an important intra and inter-individual variability. Lipid values tested before two years of age often do not represent correctly the real lipid profile of the subject, as lipid values often stabilise afterwards. Moreover, a dietetic treatment is not advisable before two years of age, as an unnecessary lipid restriction may lead to brain and development delay [10,20]. Lipid profile evaluation prior to two years of age should be performed only in specific cases, for example if HoFH is suspected, and this decision must be taken by a specialized lipid centre. Lipid values after ten years of age might be altered by pubertal activation, therefore this is not an advisable age for screening [10,31]. Detection of a pathogenic mutation in a child is the gold standard for the diagnosis of FH and it is performed with Next Generation Sequencing (NGS). Sitosterolaemia, a very rare disorder, may mimic FH in childhood. Secondary causes of hypercholesterolaemia must be excluded [7].

## 5. Risk Stratification

Lifelong elevation of cholesterol levels on a genetic basis imposes substantially greater risk than acquired hyperlipidaemia in midlife. The risk of early CHD in individuals with FH is associated with lifelong exposure to elevated LDL-C levels; individuals with FH who are untreated have 20-fold increased risk of premature CHD, but fortunately such an outcome can be avoided [32]. A variety of predictor variables have been proposed: genetic risk scores, clinical, biochemical, inflammatory markers [33], image parametres (IMT, coronary artery calcium score) [34,35]; but to date, none of these predictor variables have proven to be sufficiently robust to allow widespread use in clinical practice. Traditional cardiovascular risk factors and a history of premature CHD in a parent increase the risk in the child and should always be part of the routine risk assessment for children with hypercholesterolaemia [7,10] (Table 2).

## 6. Management of Hypercholesterolaemia in Childhood

Management of children with hypercholesterolaemia can be divided into two intervention levels: first a general evaluation, aimed at detecting children at cardiovascular risk, carried out by family doctors and/or through screening programs, then a specific evaluation of children at high cardiovascular risk and/or with severe hypercholesterolaemia, carried out by specialized lipid centres. Cholesterol and triglycerides levels (considered in a fasting profile) can be divided into acceptable, borderline or high (Table 3) [12].

**Figure 2:**
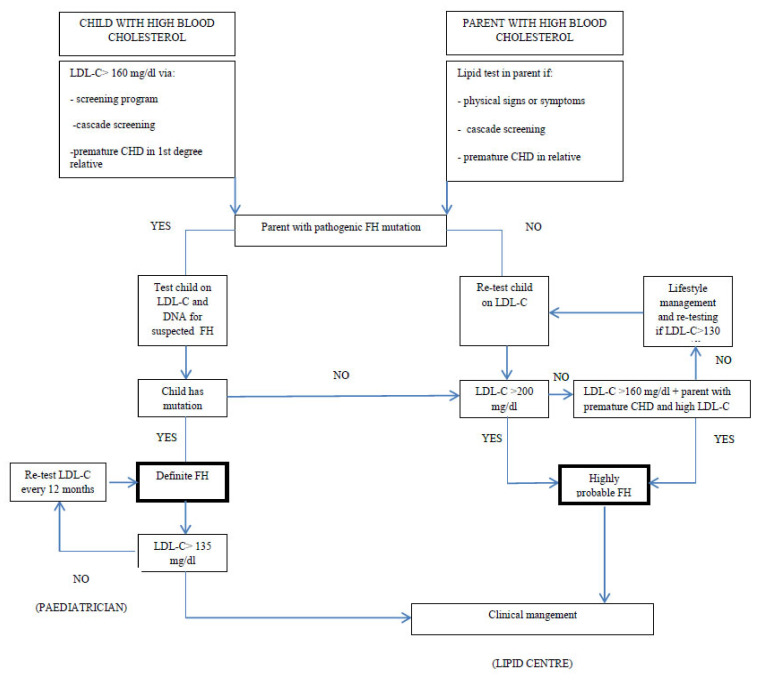
Diagnosis of FH in childhood, modified from Wiegman et al. [7].

Collecting a problem-oriented family history, aimed at detecting cardiovascular events, is of utmost importance when approaching a child or adolescent with hypercholesterolaemia. A cardiovascular-disease-tailored family history collection is easy to do, easy to reproduce and must be carried out by any health professional dealing with children with hypercholesterolaemia. Cardiovascular events (angina, heart stroke, brain stroke) are considered premature if they occur before 55 years of age in men or before 60 years in women; later on, they cannot be considered as premature. The presence of dyslipidaemia in first and second degree relatives must be evaluated as well. The first biochemical report of hypercholesterolaemia (30) must always be confirmed with a second blood sample collected after a twelve-hour night fasting, which may be performed even after a few months. The result must always be analysed taking into account age, sex and pubertal status of the subject. Secondary causes of hypercholesterolaemia, such as hypothyroidism, kidney disease, liver disease, obesity, anorexia nervosa, and/or pharmacological treatments, must always be excluded. This first step management of children with hypercholesterolaemia is not expensive and represents an easy tool for family doctors, who can perform such analyses during routine medical controls. Hypercholesterolaemia in a subject with a positive family history for cardiovascular disease and/or hypercholesterolaemia must always be studied with further analyses. Management of children with hypercholesterolaemia according to their lipid values and to their family history is summarized in Figure 2, adapted from EAS Consensus [7].

First of all, children with hypercholesterolaemia and positive family history for cardiovascular disease and/or positive family history for hypercholesterolaemia evaluated at the first visit in a specialized lipid centre, are administered an anamnestic CHD-oriented questionnaire. Written blood lipid profile of both parents is requested. Often parents refer pathological lipidaemic values as normal [24], therefore precise values have to be seen by the child doctor. Afterwards, blood analysis is performed. The usual blood panel used in specialised lipid centres include evaluation of complete lipid profile, Apolipoprotein A1, Apolipoprotein B, lipoprotein(a), thyroid markers, total homocysteine, genetic analysis for mutations of LDL-R gene and other genes involved in FH phenotype. Dietary habits are evaluated using a food frequency questionnaire (FFQ). Before the FFQ is analyzed, general nutritional claims are illustrated. When the FFQ has been analyzed, specific and tailored nutritional and dietary advice is given. Six months later, the patient is usually re-evaluated. During this second visit the diagnosis is expressed in more detail and therapeutical and follow up managements are explained and discussed with the patient and his or her family. At least six months of dietary and lifestyle treatment are needed before starting any possible pharmacological treatment. Pharmacological treatment must not substitute dietary and lifestyle recommendations; on the contrary, these two therapies are complementary. Dietary intervention must be carefully tailored on the child’s and family’s needs and beliefs. Parents who experienced CHD or hypercholesterolaemia often tend to convince their children to follow their own diet. This is a very dangerous habit. Children are not “small adults”, they are still growing, so it fundamental that they receive enough macronutrients and micronutrients to enable a correct physical and neurological development. This is why nutritional advice must be given by an expert paediatric lipidologist, so as to ensure an adequate development and to maintain a low lipid intake. Pharmacological therapy is proposed according to international guidelines and it is discussed with both parents and with the patient, in order to obtain the best possible compliance [10]. Then, patients attend visits every six months, to monitor growth and adherence to the nutritional and/or pharmacological prescriptions.

## 7. Treatment of Hypercholesterolaemia in Childhood

### 7.1. Nutritional and Lifestyle Treatment

Nutritional advice and promotion of a healthy lifestyle are the milestones of treatment of hypercholesterolaemia in childhood. From a nutritional point of view, the aim of the treatment is to teach the child and his or her family correct nutritional habits that can last until adulthood and thereafter. The intake of saturated fatty acids rich foods has to be limited, as saturated fatty acids account for a possible increase in blood cholesterol levels. A low lipid diet is recommended, including less than 30% of total daily energy from lipids, less than 7% of total daily energy from saturated fatty acids and a daily cholesterol intake lower than 200 mg. An adequate intake of fruit and vegetables, fish and legumes are also recommended [7]. Mediterranean Diet is an ideal model for children with hypercholesterolaemia, as it is based on high weekly intake of fish, legumes, fruits and vegetables, low intake of salt, and the use of olive oil. Steamed and baked preparations are preferred, putting a strong limitation to fried foods. The ideal diet should have the following characteristics [31]: 12–14% of total daily calories from proteins, with an animal/vegetal protein ratio of 1:1; 55–60% of total energy intake from carbohydrates, preferring complex carbohydrates with complex/simple carbohydrates ratio of 3:1; 25–30% of total daily calories from lipids, subdivided between saturated, monounsaturated and polyunsaturated fatty acids. The ideal energy intake provided by saturated fatty acids should be lower than 10% of total daily calories, with monounsaturated fatty acids providing between 10% and 15% of total daily calories and polyunsaturated fatty acids 5–10%. Food intake should be ideally subdivided in 5 daily meals: breakfast, lunch, dinner, morning break and afternoon break. Daily calories should be divided as follows: 20% from breakfast and morning break, 40% from lunch, 10% from afternoon break, and 30% from dinner. White meat should be proposed three times a week, fish 4 times, legumes 4 times, cheese once or twice times, and eggs once a week. Fish rich in omega-3 fatty acids should be preferred, such as salmon, cod, tuna, blue fish [31]. Big fish such as tuna should not be eaten more than twice per week, because they might contain high level of mercury [36]. No lipid restriction is recommended for children below two years of age, as lipid and cholesterol are fundamental for adequate growth and neurodevelopment. However, it is important to teach correct eating habits early in life, in order to avoid both dangerous excesses and lack of nutrients due to inadequate eating habits.

### 7.2. Nutraceuticals

Nutraceuticals may be considered for a short period, together with nutritional treatment, even if no evidence is available for paediatric age yet. Alimentary fibers such as psyllium, glucomannan, guar gum and oats, can lower total cholesterol and LDL cholesterol levels [37,38]. The lipid lowering effect of alimentary fiber has been validated by the European Food Safety Authority [39]. The use of phytosterol [1–2 g/day] can reduce total cholesterol levels in children with mild hypercholesterolaemia and in children with FH [40,41], but the long-term safety has been questioned [42]. Other nutraceuticals have been tested in children with hypercholesterolaemia together with dietetic approach, including red yeast rice [43,44], omega-3 and omega-6 long chain polyunsaturated fatty acids [45], soy proteins [46,47] and probiotics [48]. Red yeast rice can lower LDL-C levels inhibiting hepatic cholesterol metabolism [43,44]. Omega-3 long chain polyunsaturated fatty acids, in particular docosahexaenoic acid, act improving quantitative level of HDL cholesterol and reducing triglycerides levels [45]. Soy protein have a lipid lowering effect: blockage of bile acid and/or cholesterol absorption are possible mechanism underlying this effect, as well as the stimulation of low-density lipoprotein receptor [46,47]. Probiotics may reduce blood cholesterol levels by several mechanism, including production of short-chain fatty acids that can interfere with cholesterol biosynthesis and increase in bile acids excretion [48].

### 7.3. Lifestyle

The promotion of an active lifestyle with adequate amount of physical activity and sport is another milestone of treatment of patients with hypercholesterolaemia. Possible secondary causes of CHD risk, such as cigarette smoke, obesity, diabetes and hypertension should be ruled out. If an adequate dietetic and lifestyle approach is started before pubertal age, these habits are more likely to last during the following years [31].

### 7.4. Pharmacological Treatment

Bile acid sequestrants, such as cholestyramine, have been the only possible pharmacological treatment for hypercholesterolaemia in childhood for many years. These drugs act sequestrating bile acids. They do not cause systemic adverse effects, but they often cause gastrointestinal adverse effects such as diarrhoea and abdominal pain and they are not palatable at all, so it is difficult to obtain an optimal patient compliance. Nowadays, statin therapy is the main pharmacological treatment for hypercholesterolaemia in childhood. Statins act through the inhibition of the endogenous synthesis of cholesterol and the up-regulation of LDL-receptors in the hepatocytes. Simvastatin, lovastatin atorvastatin, pravastatin, fluvastatin and rosuvastatin are approved in childhood in Europe and in the United States. In the United States, pravastatin can be used from eight years of age, whereas all the above-mentioned statins can be used from ten years of age. In Europe, the use of rosuvastatin has been approved in children from six years of age [49]. Statin therapy in childhood has been proved to be safe. A recent study has demonstrated that the sooner statin therapy is started, the higher is the reduction of CHD risk in adulthood [50]. Statin therapy should be initiated with the lowest recommended dose, then the dose should be titrated according to LDL cholesterol levels and patient’s therapy tolerance. LDL cholesterol levels ≤ 130 mg/dL from ten years of age or a reduction of 50% of pre-treatment cholesterol levels in children of age between 8 and 10 years are recommended as a target during statin treatment [7]. Reaching this target is not always possible, so in some cases ezetimibe or bile acid sequestrants may be associated to statin therapy [15,51]. Ezetimibe acts with a selective inhibition of intestinal cholesterol absorption and is approved in childhood starting from ten years of age; it is usually well tolerated and it has few side effects. In the clinical practice, pharmacological therapy for hypercholesterolaemia for paediatric patients must always be well evaluated and discussed with the patient and his or her family. Parents often find it difficult to accept a pharmacological therapy, especially for young children. On the other hand, adolescents might not be interested in their pathology and therefore their compliance to the therapy can be very low. In severe cases, especially in HoFH, LDL-apheresis can be necessary. LDL-apheresis can be started from two years of age and must be performed in specialized centres. Lomitapide and mipomersen are two new drugs that have been recently approved for HoFH therapy. Lomitapide inhibits microsomal triglyceride transfer protein and can be assumed orally. Mipomersen is a subcutaneous drug that binds to the messenger RNA encoding for Apolipoprotein B-100 (ApoB-100), a protein that is the main component LDL and very low-density lipoprotein (VLDL); thus, the m-RNA is degraded by the enzyme ribonuclease H, and ApoB-100 is not translated [7]. Human monoclonal antibodies known as PCSK9 inhibitors (alirocumab, evolocumab and bococizumab) represent a novel group of anti-cholesterol drugs [7]. PCSK9 inhibitors have been proven to lower both LDL and Lp(a) levels [7]. Lipid-lowering therapies in childhood used in Italy are summarised in Table 4.

## 8. Follow-Up

Patients’ follow up should include paediatric clinical evaluation every six or twelve months, so as to monitor patient’s growth and adherence to nutritional, lifestyle and pharmacological therapy. In case of pharmacological therapy, its adherence and tolerance must be strictly monitored, especially in those patients that practice sport competitions and/or are under other pharmacological treatments. Statin adverse effects are rare in paediatric age, the more common being myopathies and hepatotoxicity. Therefore, liver (hepatic aminotransferases) and muscle (creatine kinase) enzymes should be measured before starting treatment and then periodically monitored [52]. When using recommended dose, the dose-response effect of statin therapy is not linear: the highest reduction in LDL cholesterol levels occurs at low dosage, then, even if the dose is doubled, a maximum reduction of 6–7% of LDL cholesterol may be obtained [53]. Therefore, the use of high dose of statins in children should be strictly evaluated, taking into account the possible adverse effects caused by a prolonged drug exposition. When LDL-C target is not reached even if high dose statin and/or multi drug therapy is used, therapy adherence and response should be monitored even more carefully. Paediatric patients with non-complicated and well controlled HeFH can be followed up by family doctors after having been diagnosed and screened in a specialized lipid centre. On the contrary, paediatric patients with very high LDL cholesterol levels, with multiple CHD risk factors, and with adverse effects due to pharmacological therapy and/or with HoFH, should be followed up in a specialized lipid centre, with a multiprofessional team including paediatricians and cardiologists. Recommendations for children and adolescents with HeFH are summarised in Table 5.

## 9. Integration of Care and Transition

**Figure 3:**
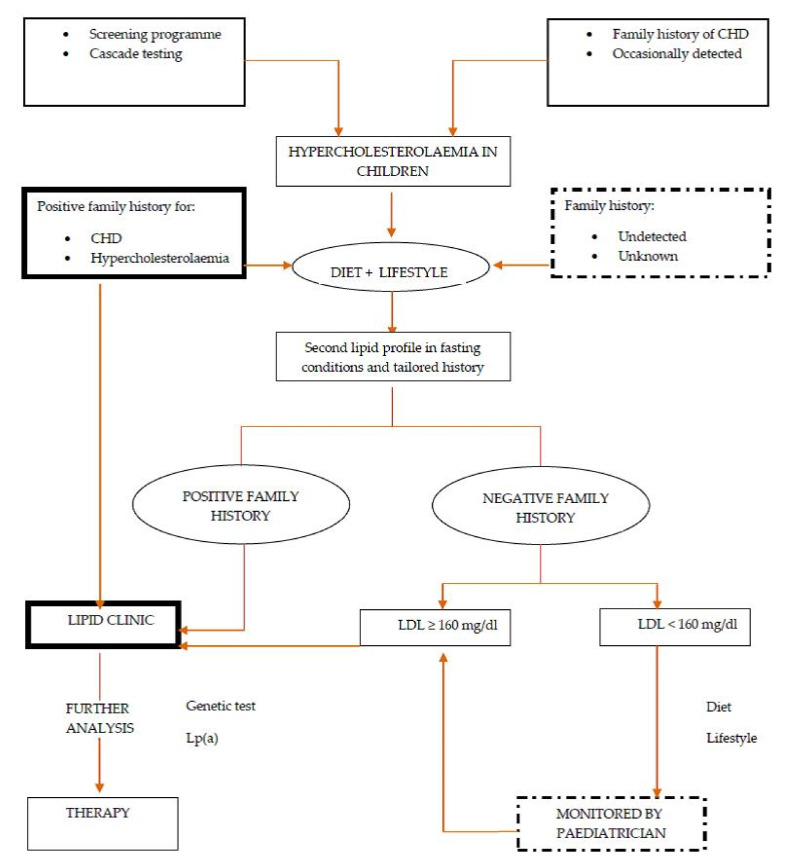
Management of hypercholesterolaemia in childhood (created by Pederiva C and Capra M.E.).

Optimal care of children and adolescents with hypercholesterolaemia requires a multidisciplinary framework integrated across primary care, paediatric specialists, and adult services [15]. Patients’ support groups and networks have a critical role in improving the care of children and adolescents: empowering patients raises the awareness of the risks and of the different possible treatments and improves collaboration between patients’ groups and the medical/scientific world [54]. Registries facilitate research and education and lead to better health outcomes for patients [25]. The transition from adolescence to adulthood is a very delicate period in terms of therapy compliance. Therefore, a good and steady nutritional and lifestyle education starting from the early stages of childhood is of utmost importance in order to obtain a long-lasting compliance [52]. Female adolescent patients must be advised that statins can be teratogenic, therefore a gynecological consultation should be provided and, when necessary, specific contraception should be prescribed. If oral contraceptives are prescribed, lipid levels should be carefully monitored, as oral contraceptives can cause an increase in LDL-C and triglyceride levels and they can have a pro-thrombotic action. When planning a pregnancy, statin therapy should be ideally stopped three months before conception and stopped during pregnancy and lactation [55]. Taking into account the therapy stop due to a possible pregnancy or lactation, statin therapy should not be delayed in female patients. Transition from paediatric to general lipidologist should be started at 16–18 years of age, ideally when puberty is completed [53]. Sometimes this transition can be seen as “childhood end” by parents, who often ask to keep on been followed up by paediatric lipidologist. On the other hand, when parents with hypercholesterolaemia are followed up in a specialized lipid center, they are more prone to let their children be followed up by a general lipidologist. In this context, a network connecting all professional figures involved in the management and treatment of patients with hypercholesterolaemia is really essential. A summary of management of hypercholesterolaemia in childhood can be found in Figure 3.

## 10. Conclusions

Early detection and treatment of patients at high CHD risk and/or with hypercholesterolaemia is a fundamental milestone in the atherosclerosis prevention pathway. In the last ten years, many screening strategies involving the whole family have been carried out: selective screening, cascade screening, reverse screening, universal screening. The aim of all these screening strategies is to identify and treat these patients. Unfortunately, the knowledge of the epidemiological and clinical importance of atherosclerosis and its implication is still low, especially when dealing with paediatric patients [56]. The knowledge of the importance of detecting and treating hypercholesterolaemia in childhood is still even lower, both among paediatricians and the general population. The presence of specialised networks, such as LIPIGEN, enables exchange of information and sharing of guidelines, thus enhancing the detection of patients with severe hypercholesterolaemia, with a better clinical management. Paediatricians, general lipidologists, patients’ organizations and politicians must strictly collaborate in order to develop a health standard policy for paediatric patients at high CHD risk and for their families.

## Figures and Tables

**Table 1:** Screening strategies for FH in children and adolescents.

Universal screening: population screening for a specific age group Selective screening: screening for a specific (high-risk) population Cascade screening: from an index case (parent) to family members (including children) Reverse screening: from an index case (child/adolescent) to other family members Child-parent screening: from children screened at a specific age to parents

**Table 2:** Optimizing diagnosis of FH in children and adolescents.

The most important key selective screening points are positive family history for premature CHD and elevated LDL-C levels. Phenotypic diagnosis should be made using blood cholesterol testing LDL-C > 190 mg/dL on two different blood samples performed at baseline and after a three-months period of nutrition and lifestyle treatment is highly suggestive of a diagnosis of FH. LDL-C > 160 mg/dL and a positive family history of premature CHD in first degree relative and/or high blood cholesterol in first degree relative indicates a highly probable diagnosis of FH. LDL-C > 130 mg/dL and a parent with genetic diagnosis of FH is indicative of probable FH Secondary causes of hypercholesterolaemia should be ruled out DNA testing is the gold standard of the diagnosis. When a pathogenic LDL-R mutation is found in a first degree relative, children and/or adolescent should also be genetically tested. In case of a parent’s death for CHD, a child with hypercholesterolaemia (even if mild) should be tested genetically for FH and Lp(a) levels should be assayed.

**Table 3:** Cholesterol and triglycerides levels considered as acceptable, borderline or high in childhood [12].

	Acceptable	Borderline	High
**Total cholesterol (mg/dL)**	<170	170–199	≥200
**LDL-cholesterol (mg/dL)**	<110	110–129	≥130
**Triglycerides (mg/dL)**			
**0–9 years**	<75	75–99	≥100
**10–19 years**	<90	90–129	≥130
**Non HDL-cholesterol**	<120	120–144	≥145
	**Acceptable**	**Borderline**	**Low**
**HDL-cholesterol**	≥45	40–44	<40

**Table 4:** Lipid-lowering agents for the treatment of FH in children/adolescents.

Lipid Lowering Agent	Mechanism of Action	HeFH Children	HoFH Children
**statins**	competitive inhibition of 3-hydroxy-3-methylglutaryl coenzyme A (HMG-CoA) reductase	from 10 years (8 years for pravastatin)	in association with ezetimibe/other lipid lowering drugs
**bile acid sequastrants**	inhibition of intestinal cholesterol absorption	from 8 years (fat soluble vitamins supplementation)	in association with statins/other lipid lowering drugs
**ezetimibe**	selective inhibition of intestinal cholesterol absorption (inhibition of the sterol transporter Niemann-Pick C1L1)	from 10 years	in association with statins/other lipid lowering drugs
**lomitapide**	inhibits microsomal triglyceride transfer protein (MTP)	approved for the treatment of HoFH in adults (EMA-AIFA)	in clinical trials for HoFH (NCT04681170)
**mipomersen**	antisense oligonucleotide against the coding region of apolipoprotein B mRNA	approved for the treatment of HoFH in adults (FDA-not EMA)	not approved
**PCSK9 inhibitors**	prevent the breackdown of LDL receptors	approved for the treatment of FH in adults (EMA-AIFA)	in clinical trials for HoFH (NCT03510715) and HeFH (NCT03510884)

**Table 5:** Recommendations for management of children and adolescents with HeFH.

HeFH must be diagnosed as early as possible, so as to “gain decades of life” A late diagnosis of FH leads to a considerable reduction in the duration and in the quality of life Genetic diagnosis of FH is important for awareness of the early start of the atherosclerotic process, in order to obtain a greater adherence to the therapy and as an important knowledge for future offsprings. Positive family history for premature CHD is a very important risk factor, but it fades out if a prompt and adequate treatment is started. Analysing family history for CHD including second degree relatives may be a good suggestion. Clinical signs and symptoms of HeFH are not common in paediatric ages, except for Achilles tendon pain. Nutritional and lifestyle treatment must be started in the earliest stages of life and must be well established before puberty Smoking habit must be strictly discouraged In case of HeFH, statin therapy is available from 8 years of age. For patients with HoFH, statin therapy must be started as early as possible. Statin therapy lasts lifelong, therefore it is important to stress its safety, both for clinical health and for therapy adherence. Therapy should be started as early in girl as in boys, considering that statin therapy must be discontinued in case of pregnancy and/or lactation. If therapeutical target is not reached, adding a second pharmacological treatment might be necessary.

## Data Availability

Not applicable.

## Abbreviations

APOB	Apolipoprotein B
APOB-100	Apolipoprotein B-100
CHD	Coronary Heart Disease
cIMT	Carotidal Intima Media Thickness
EAS	European Atherosclerosis Society
FFQ	Food frequency questionnaire
FH	Familial Hypercholesterolaemia
HDL-C	High Density Lipoprotein Cholesterol
HeFH	Heterozygous Familial Hypercholesterolaemia
HoFH	Homozygous Familial Hypercholesterolaemia
LDL-C	Low Density Lipoprotein Cholesterol
LDLRAP1	Low Density Lipoprotein Receptor Adaptor Protein 1
LIPIGEN	Lipid transport disorders Italian Genetic Network
Lp(a)	Lipoprotein(a)
NLA	National Lipid Association
NGS	Next Generation Sequencing
PCSK9	Proprotein convertase subtilisin/kexin type 9
PDAY	Pathobiological Determinants of Atherosclerosis in Youth
VLDL	Very low-density lipoprotein
